# Psoriasiform mycosis fungoides–cutaneous T-cell lymphoma in an African American

**DOI:** 10.1016/j.jdcr.2023.05.035

**Published:** 2023-06-05

**Authors:** Austinn C. Miller, Alexzandra Mattia, Anthony Thompson, Laurie A. Temiz, Susuana Adjei, Stephen K. Tyring

**Affiliations:** a Dermatology Associates of Tallahassee, Tallahassee, Florida; b Florida State University College of Medicine, Tallahassee, Florida; c Centers for Clinical Studies, Webster, Texas; d Meharry Medical College, Nashville, Tennessee; e Department of Dermatology, University of Texas Houston, Houston, Texas

**Keywords:** cutaneous T-cell lymphoma, CTCL, mycosis fungoides, MF, skin of color, psoriasis

## Introduction

Cutaneous T-cell lymphomas (CTCLs) are a group of neoplasms characterized by monoclonal proliferation of skin-homing T cells that show considerable variation in clinical presentation, histologic appearance, immunophenotype, and prognosis.^1^ CTCL is often divided into mycosis fungoides (MF)-CTCLs and non–MF-CTCLs. MF-CTCL is a rare disease, occurring in approximately 0.5 per 100,000 individuals in the United States.^2^ Despite the rate of MF-CTCL being highest among African Americans, there is a lack of clinical images demonstrating this condition in skin of color. Here, we describe a case of psoriasiform MF-CTCL misdiagnosed as psoriasis in an African American patient and provide excellent teaching images. Consent for publication of all patient photographs and medical information was provided by the authors at the time of article submission to the journal stating that all patients gave consent for their photographs and medical information to be published in print and online, with the understanding that this information may be publicly available.

## Case Report

**Fig 1:**
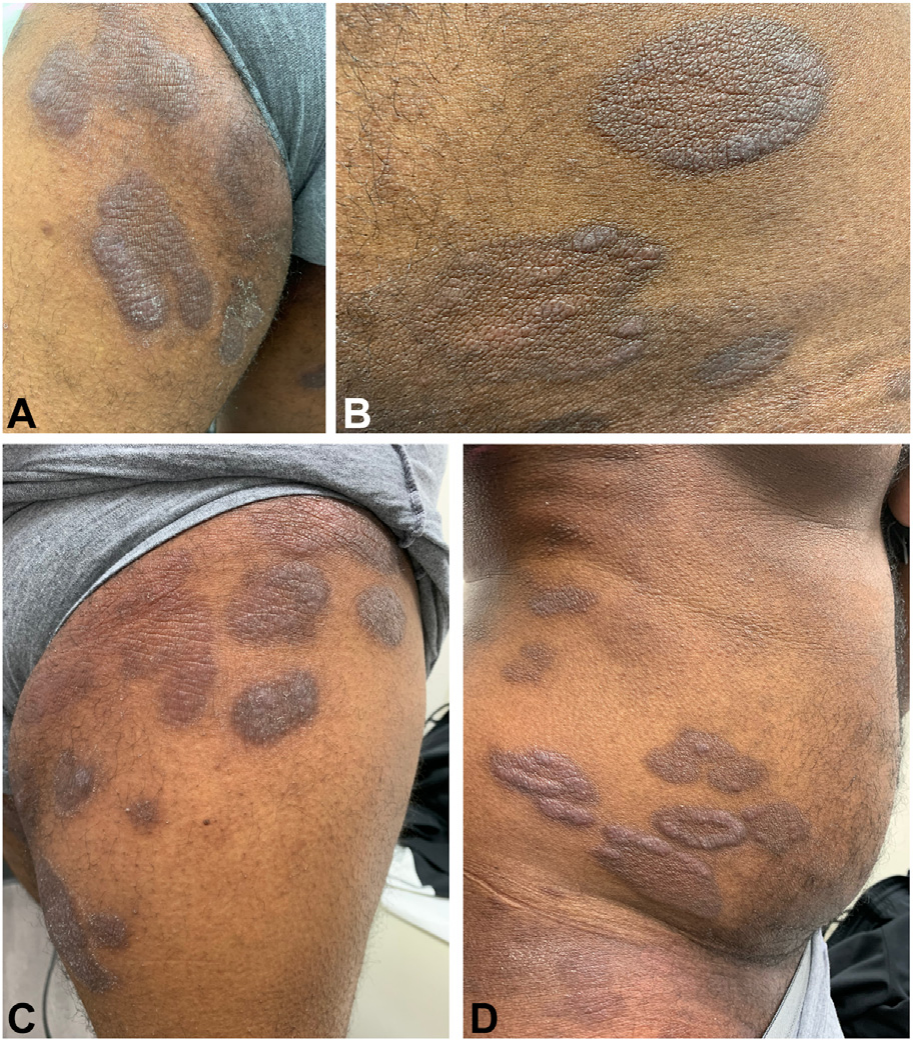
**A,** Plaques with varying degrees of hyperpigmentation and fine scale, located on the right medial thigh. The thickness of scaling in this image is reduced secondary to recent topical corticosteroid use. **B,** Admixture of pigmentation within each plaque. **C,** Arcuate lesions with fine scale and admixture of pigmentation. Prominent lichenification is present on the proximal lesions. **D,** Arcuate lesions with scale and patches and plaques.

A 45-year-old African-American man presented with well-circumscribed plaques, exhibiting fine scale on his back, abdomen, groin, and axilla that had progressively increased in size and number over the last 3 years (Fig 1). The lesions first appeared in his right groin, and within 2 weeks, the lesions had spread to his back. Besides pruritus, the patient had no other symptoms. Six months after the rash appeared, a biopsy was performed, which returned with indeterminate results. Given the characteristic scaly plaques, the patient was diagnosed with plaque psoriasis by a dermatologist and was treated with apremilast. Due to lack of improvement and side effects, the medication was discontinued. The patient received no treatment over the next 6 months, and the plaques spread to his left groin, abdomen, and bilateral axilla. The patient was prescribed guselkumab and remained on this medication for 6 months, noting that the plaques continued to increase in number, become scalier, and develop fissures. The patient was then switched to secukinumab but experienced similar results and the medication was discontinued.

Given the lack of improvement on psoriasis treatments, Wood’s lamp examination was performed, and fluorescence was detected on his skin lesions. The patient was prescribed terbinafine, antifungal spray, and shampoo/body wash. The patient noted decreased scale within 2 weeks of treatment initiation. After 6 weeks, the patient began experiencing refractory pruritus and developed more lesions. The antifungal medications were discontinued, and the patient was started on both oral/topical steroids and hydroxyzine.

**Fig 2:**
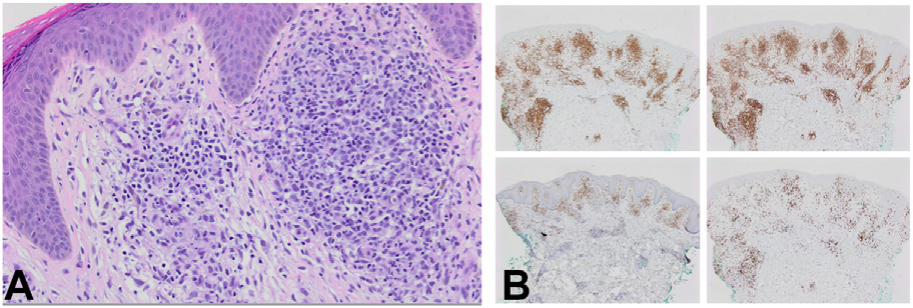
**A,** Band-like papillary dermal lymphoid infiltrate composed of small- to medium-sized hyperchromatic lymphocytes mixed with histiocytes and plasma cells. (H&E 200×) **B,** Lymphocytes are predominantly composed of T cells (CD3+), with a CD4:CD8 ratio of approximately 4:1 and loss of CD7 in the CD4+ lymphocytes. (Top left: CD3 stain 40×, top right: CD4 stain 40×, bottom left: CD7 stain 40×, bottom right: CD8 stain 40×). *H&E*, Hematoxylin and eosin.

After 3 years of failed immunosuppressive and antifungal treatments from a multidisciplinary team of primary care physicians and dermatologists, the patient was referred to our institution. A detailed history and physical examination was performed. Given the treatment-resistant nature of the psoriasiform-appearing rash, another biopsy was performed (Fig 2). Biopsy revealed dermal lymphoid infiltrate with small- to medium-sized hyperchromatic lymphocytes, mixed with histiocytes and plasma cells. Lymphocytes were predominately CD3+, with a CD4:CD8 ratio of 4:1. The CD4+ lymphocytes did not react with CD7. Histological findings, in conjunction with a T-cell receptor beta gene rearrangement, were consistent with MF-CTCL. The patient was referred to MD Anderson Cancer Center for further treatment.

## Discussion

Over 20 variants of MF-CTCL have been documented on the basis of distinctive clinicopathologic features, clinical behavior, and/or prognosis.^1^ Many patients have nonspecific forms that may mimic other dermatoses (ie, chronic eczema or atopic dermatitis) with nondiagnostic biopsies. Rarely, MF-CTCL presents with psoriasiform plaques. Although the exact incidence is unknown, 1 study of 436 MF-CTCL patients reported psoriasiform variant frequency as 4.7%.^3^ Of the 8 case reports published on psoriasiform MF-CTCL misdiagnosed as psoriasis, this is the first documented in an African American.^4^

In skin of color, 3 common and often intertwined presentations of MF-CTCL usually occur.^5^ First, dyspigmentation may result in a mixture of asymptomatic hypopigmented and hyperpigmented lesions (Fig 1, Fig 2, *A-D*).^5^ In addition, some lesions may mimic a more common dermatosis, such as tinea, psoriasis, atopic dermatitis, vitiligo, or lichen planus.^5^ The third presentation involves pruritus and secondary lichenification and hyperpigmentation, which often masks characteristic MF-CTCL clues (Fig 1, *C*).^5^ Classic MF-CTCL morphology in skin of color includes arcuate lesions with scale and poikilodermatous patches and plaques with atrophy (Fig 1, Fig 2, *A-D*).^6^ The minimum size of MF-CTCL is generally 5 cm, whereas many of the histologic mimics are smaller lesions.^5^

In psoriasiform MF-CTCL, clinical presentation includes thick, scaly, well-demarcated, erythematous psoriasiform plaques, which are difficult to differentiate from psoriasis clinically.^6^ More specific clues to psoriasiform MF-CTCL include accompanying alopecia, ulcerative lesions, and lymph node enlargement. Distribution may also be helpful. Classic MF-CTCL occurs in the “swimsuit” distribution (buttocks, breasts, and intertriginous regions), whereas psoriasis most commonly occurs on the scalp and extensor surfaces.^5^ In contrast to psoriasis, psoriasiform MF-CTCL may demonstrate epidermotropism with infiltration of abnormal lymphocytes.^6^ Gene rearrangement studies may also help differentiate both; however, inadequate samples may produce false negative results.^6^ Biopsies from different anatomic sites that show monoclonal T cells are also helpful in establishing a diagnosis in indeterminate cases.^7^

Correct and timely diagnosis is crucial, given psoriasis treatment may exacerbate MF-CTCL. Most cases of exacerbation following immunosuppressive treatment have occurred secondary to cyclosporine, methotrexate, or tumor necrosis factor-α inhibitors. A review of patients who developed MF after systemic or biological therapy for psoriasis revealed 30 cases.^8^ Of these, 25 patients were treated with a tumor necrosis factor-α inhibitor^8^ and 6 patients were treated with either an IL-17 or IL-23 inhibitor.^8^

Staging is important because it directs management and carries prognostic significance. Although diagnosis of MF-CTCL is typically prolonged and certain treatments may exacerbate disease, it rarely progresses beyond the plaque stage in many patients. Approximately 10% of patients develop nodules or tumors.^5^ However, African Americans have a significantly increased risk of presenting with a higher T-stage (T3-T4) and a higher rate of mortality compared with Whites.^9^ The reason for higher stage at presentation is unknown but inability to recognize signs and symptoms of MF-CTCL and differentiate it from other conditions in those with skin of color likely contributes. Regardless, given the potential for poor clinical outcomes relating to higher disease stage, this is concerning.^9^

The key to early treatment is a timely diagnosis. Although MF-CTCL’s qualities as a mimic cause confusion, it is important to keep it on the differential, especially in patients diagnosed with other conditions refractory to treatment. Moreover, special attention should be paid to MF-CTCL in skin of color presenting with classic features. Biopsy should be considered, especially if previously indeterminate. This has the potential to reduce burden associated with incorrect treatments and disease progression and ultimately improve outcomes.

## Conflicts of interest

None disclosed.
